# An Emergence Framework of Carcinogenesis

**DOI:** 10.3389/fonc.2017.00198

**Published:** 2017-09-14

**Authors:** Elizabeth A. W. Sigston, Bryan R. G. Williams

**Affiliations:** ^1^Department of Otorhinolaryngology, Head & Neck Surgery, Monash Health, Melbourne, VIC, Australia; ^2^Department of Surgery, Monash Medical Centre, Monash University, Melbourne, VIC, Australia; ^3^Hudson Institute of Medical Research, Melbourne, VIC, Australia; ^4^Department of Molecular and Translational Science, Monash University, Melbourne, VIC, Australia

**Keywords:** emergence, systems biology, carcinogenesis, thermodynamics, chaos, entropy, fractals, translational research

## Abstract

Experimental paradigms provide the framework for the understanding of cancer, and drive research and treatment, but are rarely considered by clinicians. The somatic mutation theory (SMT), in which cancer is considered a genetic disease, has been the predominant traditional model of cancer for over 50 years. More recently, alternative theories have been proposed, such as tissue organization field theory (TOFT), evolutionary models, and inflammatory models. Key concepts within the various models have led to them being difficult to reconcile. Progressively, it has been recognized that biological systems cannot be fully explained by the physicochemical properties of their constituent parts. There is an increasing call for a ‘systems’ approach. Incorporating the concepts of ‘emergence’, ‘systems’, ‘thermodynamics’, and ‘chaos’, a single integrated framework for carcinogenesis has been developed, enabling existing theories to become compatible as alternative mechanisms, facilitating the integration of bioinformatics and providing a structure in which translational research can flow from both ‘benchtop to bedside’ and ‘bedside to benchtop’. In this review, a basic understanding of the key concepts of ‘emergence’, ‘systems’, ‘system levels’, ‘complexity’, ‘thermodynamics’, ‘entropy’, ‘chaos’, and ‘fractals’ is provided. Non-linear mathematical equations are included where possible to demonstrate compatibility with bioinformatics. Twelve principles that define the ‘emergence framework of carcinogenesis’ are developed, with principles 1–10 encapsulating the key concepts upon which the framework is built and their application to carcinogenesis. Principle 11 relates the framework to cancer progression. Principle 12 relates to the application of the framework to translational research. The ‘emergence framework of carcinogenesis’ collates current paradigms, concepts, and evidence around carcinogenesis into a single framework that incorporates previously incompatible viewpoints and ideas. Any researcher, scientist, or clinician involved in research, treatment, or prevention of cancer can employ this framework.

## Introduction

Consideration given to paradigms that provide the framework for the understanding of carcinogenesis by physicians, particularly surgeons, in every day practice of managing and treating cancer is minimal. Yet it is these frameworks that drive cancer research, translational research, drug development and ultimately treatment options, and impact on where research dollars are allocated (1, 2).

The predominant traditional model of cancer, the somatic mutation theory (SMT), in which cancer is considered a genetic disease (3–7), has been widely accepted as factual, rather than theory (1, 4, 5). This narrows avenues of new research and directs the way in which data is analyzed (6). While there are a number of models of carcinogenesis, these have evolved through the interaction of several disciplines and can be grouped into two main theories: those that consider cancer to be a genetic disease, including SMT, multistage models, and evolutionary models that consider the development of cancer to be due to temporal changes related to variation and selection and place genetic modifications with alteration in phenotype as the key driver of carcinogenesis (8–11), and those that consider cancer to be an issue of tissue organization, such as tissue organization field theory (TOFT) and inflammatory models (11–14). Experimental evidence can be found to support and dispute most theories, but there is no single unified theory to bring it all together. The principle underlying postulates of SMT and TOFT, as outlined in Tables 1 and 2, render them incompatible (5–7, 15).

**Table 1:** Somatic mutation theory.

Cancer is a disease of genetic mutation. Cancer is derived from a single somatic cell. The initiation process, and therefore, the process of carcinogenesis, is irreversible. The default state of a cell is quiescence. Adjacent tissue has only a supporting role in carcinogenesis. A reductionism philosophy: the properties of the whole can be inferred, deduced, calculated, and predicted from the properties of the parts. Phenomena occurring at one level (cancer at tissue level) can be explained by understanding the properties at a lower level (molecular and chemical properties at the cellular level). There is unidirectional upward causation.

**Table 2:** Tissue organization field theory.

Cancer is a disease of tissue organization comparable to organogenesis: carcinogenic agents destroy normal tissue architecture, interfering with normal cell–cell communication. The default state of the cell is proliferative with variation and motility. Carcinogenesis is reversible. A holistic or antireductionist philosophy: phenomena occurring at one level cannot be explained by understanding the properties at a lower level. There is unidirectional downward causation: genetic mutations and altered biochemistry are a result of disrupted tissue organization.

Numerous authors have identified a need for a ‘systems’ approach to cancer (15–22).

This paper presents a comprehensive framework for carcinogenesis. Through incorporating the concepts of ‘emergence’, ‘systems’, ‘thermodynamics’, and ‘chaos’, a single integrated framework for carcinogenesis has been developed, enabling existing theories to become compatible as alternative mechanisms, facilitating the integration of bioinformatics and providing a structure in which translational research can flow from both ‘benchtop to bedside’ and ‘bedside to benchtop’ (5, 7, 15, 20, 23, 24).

‘An emergence framework of carcinogenesis’ is based on extensive study of the works of numerous philosophers, researchers, scientists, and writers, including Mario Bunge (20) (physicist, philosopher, and philosopher of science) and Denis Noble (25) (biologist, physiologist, Emeritus Professor University of Oxford). The central proposition is a change in the way cancer is investigated, managed, and treated, by considering ‘cancer’ as an ‘emergent system’. This clear and well-defined framework allows integration of progress and discoveries made to date regarding carcinogenesis and cancer, and provides alternative ways to view that and new knowledge, driving the progress of research toward making an impact at the place that truly matters, the bedside.

## An Emergence Framework

### Background

‘Emergence’ is about the properties of wholes compared to those of their parts. It refers to complex systems having properties (components, patterns, or processes) that their constituents or precursors in isolation do not have. The new property is more than simply a combination of the properties of its pieces, meaning there is no simple mathematical model that explains this new property. It is a qualitative, not quantitative measure. An emergent property may (ontological) or may not (epistemological) be predictable through understanding the properties of its components. Emergent phenomena are found across all areas of study, including physics, chemistry, biology, sociology, psychology, economics, and IT, and led to the development of the field of quantum physics (20, 26–29) and the concept of the chemical reaction network theory in the study of proteins (30).

Thermodynamics is a physical theory that describes a system in terms of the thermodynamic properties (heat and temperature in relation to energy and work) of the system or its parts. Thermodynamics makes no assumption about the microscopic nature of the system; it describes the macroscopic properties and remains correct even if the microscopic assumptions about a system are proved wrong (29, 31).

Biological, or living, organisms are open thermodynamic systems that have acquired complexity through non-linear self-organizational processes and defy the second law of thermodynamics by mechanisms of metabolism. These properties cannot be deduced from molecular biological and genetic knowledge alone (20, 25, 29, 31).

Somatic mutation theory is based on the classic form in which biological systems have been described assuming that it should be possible to reconstruct complex living systems from the bottom up, starting with raw DNA code (22, 32). However, complexity in biological systems, as demonstrated by self-organizational studies and the Human Genome Project, does not require complexity at the level of the genome (32). Complexity is achieved by the repeated application of simple rules by large units (25, 29). An attempt to apply the non-linear mathematics of complexity to understand the combination of gene interactions to generate a single function in a genome of 30,000 genes, as an example, would yield 2 × 10^72403^ possible combinations (33). Therefore, to understand cancer only as a genetic disease is to underestimate its complexity (32–34).

Somatic mutation theory reasoning leads to the concept that the ultimate causative level is the most microscopic one, the molecular level of genes. This has caused attention to be focused on a level that does not enable an understanding of cancer as an emergent complex system (17). This does not mean that genetics, molecular biology, and immunology have not contributed enormously to advances in the understanding of the biology of cancer (1, 17). This has led to the identification of therapeutic targets, development of small molecule cancer drugs, and application of immunotherapeutics. However, these advances at the same time have both colored and limited the way in which data has been interpreted. Shortcomings and paradoxes uncovered by research findings have been attempted to be addressed through modifying the model (35), rather than questioning the fundamentals (1).

Tissue organization field theory addresses many of the shortcomings of SMT (1). It moves focus away from genes as the centric cause of cancer and instead directs it to disruption of patterns of tissue organization (3, 12, 13, 36). This framework, however, implies that genetic mutations are always the result of disruption of tissue organization and play no causative role in carcinogenesis (7, 15). Brücher’s proposed paradigm places chronic inflammation as an essential key in carcinogenesis and is supported by human and animal studies (4). However, it too discounts entirely a causal role of genetic changes.

Somatic mutation theory and TOFT both assign causation to a specific level. The causation of cancer, however, involves numerous processes of a multistage nature from molecular to environmental levels (17). There is no single privileged level of causation: causation flows in both upward and downward directions (17, 22, 25, 37, 38).

A common flaw in both SMT and TOFT is the importance attached to assuming the default state of an individual cell as either quiescent or proliferative. There is ample evidence to suggest a ‘default state’ of individual cells *in vivo* does not exist. The ‘state’ of cells (quiescent or replicating) is largely shaped by their roles and positions within their community, or, as discussed later, their ‘system’ (7). As pointed out by several authors, SMT and TOFT have been made artificially incompatible; yet experimental evidence supports that both models have value (5, 7, 15).

A systems approach enables the absorption of SMT, TOFT, and other theories into a unified concept, allowing them to be compatible as contributors to a ‘cancer system’ within an emergence framework (5, 7, 15, 20, 23).

### Key Concepts

In generating an emergence framework of carcinogenesis, a basic understanding of the concepts of ‘emergence’, ‘levels’, ‘system’, ‘complexity’, ‘thermodynamics’, ‘entropy’, ‘chaos’, and ‘fractals’ is required.

#### Emergence, Levels, Systems and Complexity

An ‘emergent property’ typically refers to a property or properties possessed by a whole that its parts lack. Some philosophers argue that ‘emergence’ can only be used when the property is unpredictable or unexplainable by contemporary theories (20). Mario Bunge, a preeminent physicist and philosopher of science, identified that this is not the sense in which ‘emergence’ is used in biology or science. He provided a clear and simple definition, suitable for science, in his 1977 Treatise as follows (39):

$$\begin{array}{l} P\text{ is a global }(\text{or collective or non-distributive})\;\\ \text{property of a system of kind }K\text{, none}\\ \text{of whose components or precursors possesses }P\\ \text{where `}P'\text{ is the `emergent property' of the system} \end{array}$$

This definition is not limited by the need to be ‘unpredictable’.

**Figure 1:**
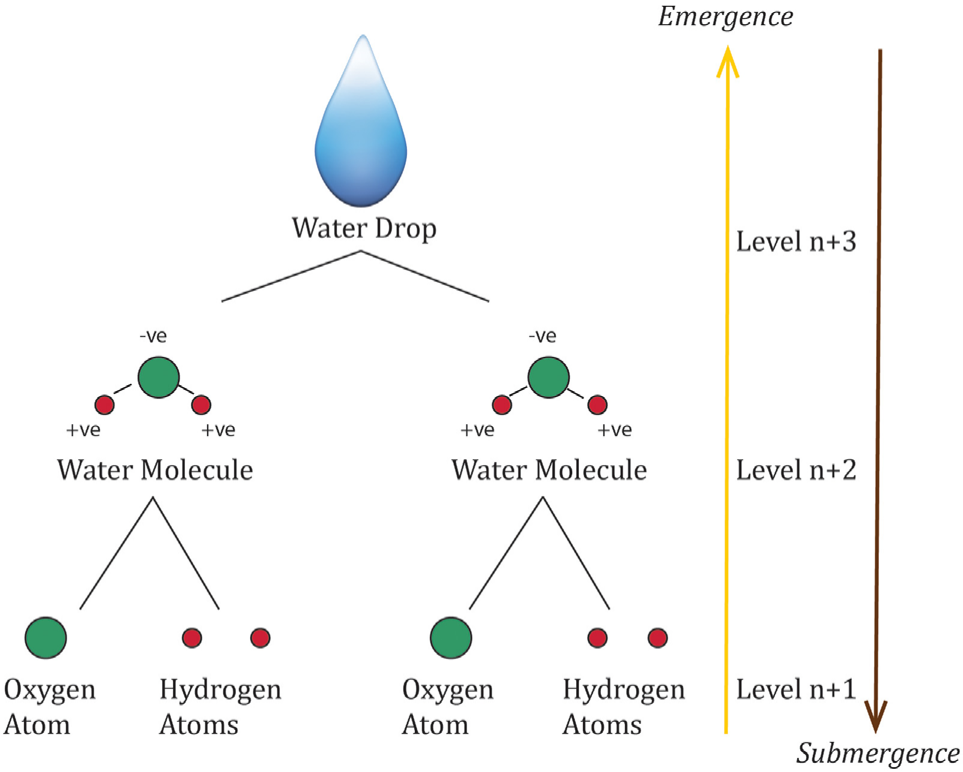
Formation of a water drop as an example of emergent hierarchical levels, with each level possessing a property or properties that is more than simply the sum of the properties of its parts.

A ‘level’ is a collection of things that have a certain property in common. Combinations of lower-level things assemble to constitute a new level, or higher-level thing. Every higher level is characterized by an emergent property, not possessed by any of the lower-level things. This implies a level hierarchy (7, 23, 32). This is demonstrated below in Figure 1, using the formation of a water drop as an example.

Level ascription is not a one-off process for any specific entity, but depends on the way one chooses to decompose the parts. This will be determined by the ‘system’ that is the subject of investigation (37).

For a complex ‘system’ to come into being, it requires (20):

- The combination of two or more precursors that combine to form a new object that is *characterized by properties its precursors do not have*. Combination, as opposed to association, requires that the original items alter in the process, the system is more stable and cohesive than a simple association, it requires energy and/or time to form, and/or is rarer than an association. A proton and electron coming together to form an atom of hydrogen, as above, is an example.
- A *bonding structure* that *enables self-organization* of the collection of relations among its components generated by the combination.
- A *mechanism*, which is a process or set of processes that *bring the emergence of a property or other process in the system as a whole*.

A ‘system’, in particular a concrete system as biological systems are, is not an isolated entity. It comes into being in some sort of surrounds or environment (20, 37). To factor in the impact of the environment on a system, structure needs to be considered in two parts: the *endostructure*, which is the collection of bonds among the system components to enable self-organization as described above, and the *exostructure*, which is the collection of bonds between the system and the environment. The exostructure then determines how the environment acts upon the system (input) and how the system acts upon the environment (output). The subset of the system members that hold direct relationship with the environment are considered the system boundary (17, 20).

A ‘system’ is dynamic in nature. Components may alter over time due to changes in external influences or internal influences. The ‘system’ itself may induce feedback loops, generating downward causation or upward causation. The ability to change is the only consistent property across all systems (20, 25).

A ‘system’ has a point of self-organized criticality. At such a point, there is a system-wide transformation, which moves a system rapidly into a new state. The ‘system’ either collapses and there is submergence of properties, or the transformation results in the emergence of a new ‘system’. The transition may be triggered by a very minor event with little significance on its own. This is a qualitative leap (7).

A system, μ, called ‘s’ at a single point in time can be defined as (20):

μ(s)={C(s), E(s), S(s), M(s)}

where C(s) = Composition: collection of all the parts of ‘s’; E(s) = Environment: collection of items, other than those in ‘s’, that act on or are acted upon by some or all of the components of ‘s’; S(s) = Structure: collection of relations, in particular bonds, among components of ‘s’ or among these and items in its environment ‘E(s)’; M(s) = Mechanism: collection of processes in ‘s’ that make it behave the way it does.

As this would require an understanding of all parts of all systems at all levels, it is not practically useful. This can be limited by focusing on the *given level* that is being studied by using the intersection or logical product:

$$\begin{array}{l} \text{C}(\text{s})\cap \text{a}={\text{C}}_{\text{a}}(\text{s})\\ \text{E}(\text{s})\cap \text{b}={\text{E}}_{\text{b}}(\text{s})\\ \text{S}(\text{s})\cap \text{c}={\text{S}}_{\text{c}}(\text{s})\\ \text{M}(\text{s})\cap \text{d}={\text{M}}_{\text{d}}(\text{s}) \end{array}$$

So that

$$\mu_{\text{abcd}}(\text{s})=\{{\text{C}}_{\text{a}}(\text{s}),{\text{ E}}_{\text{b}}(\text{s}),{\text{ S}}_{\text{c}}(\text{s}),{\text{ M}}_{\text{d}}(\text{s})\}$$

Using the water drop from above in Figure 1 as an example: μ_abcd_(s) = water drop with the emergent properties of ‘*P’* = surface tension; C_a_(s) = molecules of H_2_O; E_b_(s) = atmospheric temperature and pressure, gravity, air, or other surface the water drop is in contact with; S_c_ (s) = endogenous: the arrangement of water molecules with each other *via* hydrogen bonds that pull each molecule equally in every direction resulting in a net force of zero, =exogenous: interaction of the molecules at the surface that do not have the same molecules on each side causing them to be pulled inwards and contract to the minimal area; M_d_(s) = cohesion between water molecules being stronger than adhesion with molecules in air.

To capture the dynamics of a ‘system’, or in other words the qualitative and quantitative changes of a system over time, a state-space approach needs to also be included. The quantitative properties of a system can be combined into a single function, ‘*F’*, of the system. In a simple system that has two quantitative properties, called X and Y, which are considered to be the attributes of the system, then

F={X, Y}

A snapshot at a specific point in time, ‘t’, can then be represented by *F*(t) as follows:

F(t)={X(t), Y(t)}

This is called the ‘state function’ of the system.

A vector that describes a trajectory from this point in the ‘state space’ can then represent changes over time. The trajectory represents the *history*, ‘*H*’, of the system over a period of time, ‘T’:

H= <F(t)|t∈T>

The history is confined within a box, the ‘state space’ that represents all of the really possible states of the system as determined by the law that governs the system.

This can be demonstrated by considering the movement of a pendulum, which is a linear oscillator. The *function (F)* is swinging. It has two salient properties: *momentum (q)* and *position (p)*. The law that governs the system is that ‘energy is constant’. The potential energy related to position and the kinetic energy related to momentum must always equal the total energy of the system.

At any point in time, ‘*t*’ the state of the system can be analyzed in terms of its position and momentum. This changes over time, creating a trajectory that reflects the history, ‘*H*’. If the energy is added from an environmental source, the trajectory will alter.

An ‘event’ is represented by an ordered couple of points in the ‘state space’.

If a new property arises (emergence), the tangent of the trajectory acquires a new axis. If a property is lost (submergence), an axis is lost.

#### Thermodynamics, Entropy, Chaos, and Fractals

While thermodynamics is a physical theory that describes a system in terms of the thermodynamic properties of the system or its parts, it makes no assumption about the microscopic nature of the system; it describes the macroscopic properties and remains correct, even if the microscopic assumptions about a system are proved wrong (29). Moreover, it should be recalled that total energy in a closed system is constant and energy of any system can be ordered and available for use, or disordered and unable to be used by the system (40).

Living systems are open systems and defy the second law of thermodynamics (the universe is constantly aiming toward a state of maximal entropy or thermodynamic equilibrium), by interacting with and acquiring energy from their environment. This enables the development of complexity. If their ability to acquire energy from the environment is reduced, energy is gradually dissipated as heat to surroundings and complexity is lost, as this requires energy to be maintained, and the system will degenerate. This is the process that occurs in aging and death (18, 29, 40).

New disciplines, such as non-linear dynamics (‘chaos theory’) and fractal geometry, have brought new tools and perspectives into pathophysiology. Many physiological systems are highly complex networks, with numerous recursive feed-back and feed-forward circuits, and thus they may be especially prone to develop chaotic behaviors and display fractal structures. Non-linear dynamics and fractal models have been increasingly applied in physiology and medicine (41).

**Figure 2:**
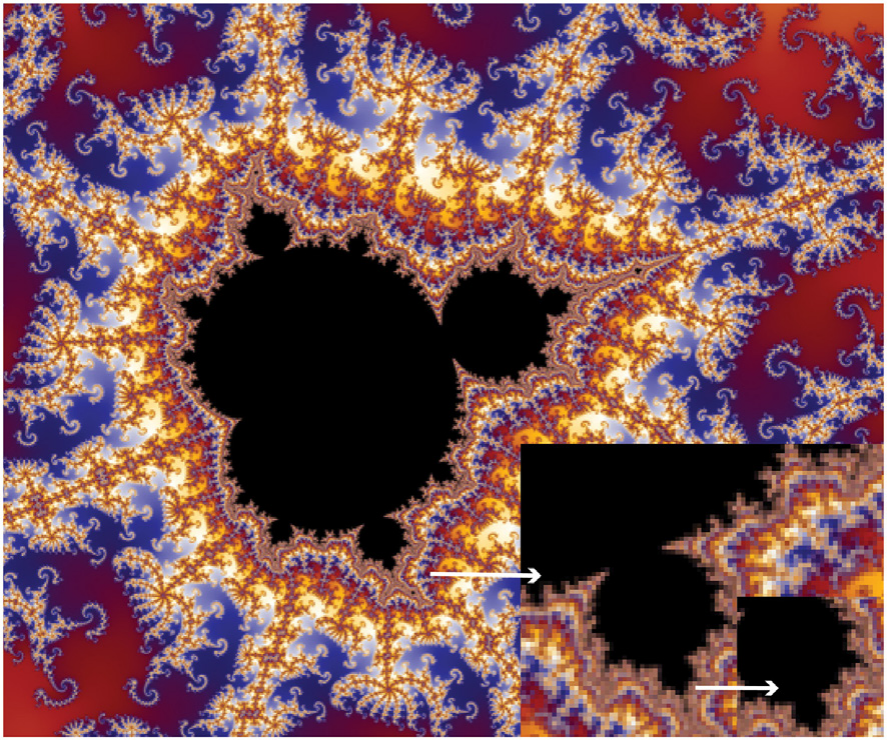
A pattern created by the Mandelbrot set exhibits an elaborate and infinitely complicated boundary that reveals progressively ever-finer recurring detail at increasing magnifications, indicated by the white arrows. The ‘style’ of this repeating detail depends on the region of the set being examined. The set’s boundary also incorporates smaller versions of the main shape, so the fractal property of self-similarity applies to the entire set and not just to its parts.

Fractals are spatial structures and have the properties of self-similarity (consists of miniature copies of itself at different levels of magnification) and/or fractal dimensions (the parameter of an object that displays how much space it occupies which is, unlike Euclidean geometry, not a whole dimension but a non-integer, the value of which is a measure of complexity), and/or rough outlines and infinite length (41–46). Fractals enable the creation of complex shapes. Nature and biological systems have mastered fractals to achieve complexity, adaptability, and efficiency. Fractal patterns can be seen in mountains, coast lines, cloud formation, branching trees, and in the human body in the branching of blood vessels, neural synapses, and lung bronchioles (41–43). One of the most well-known fractal patterns is the Mandelbrot set (Figure 2) (18, 40, 46). Fractal geometry has been noted to emerge on the surface of human cervical epithelial cells during progression to cancer (47) and observed in lymphoma and leukemia cells correlating with their biological features (48).

Chaotic dynamics in a system is a function of time (41). Chaos and complexity share the property of non-linear dynamics (18, 46, 49). A complex system keeps its non-linear processes under control through oscillations, which provide the ability to restore and maintain its steady state within its environment. There is increasing evidence that biological oscillations or ‘clocks’ and molecular motors that cycle ATP can contribute to the rise of complexity and affect morphogenesis (29, 50–53). Periodic reactions allow periodic signal transmitting between individual cells and regulate cell differentiation within an organism. Such clocks are known to be extremely stable. The predictability of their behavior implies linearity. Under certain circumstances, however, they may exhibit transition into chaotic behavior, implying unpredictability and non-linearity. As decreased usable energy moves a living system toward entropy, increased available energy pushes the system toward chaos with the possibility of creating a new initial state and the emergence of a new system (29).

Ultimately, cancer research and treatment can be progressed by shifting our focus from causal relationships that are non-linear and difficult to predict, to interpreting the patterns of cancer as an emergent system. To do this, a framework that can be employed anywhere from benchtop to bedside is likely to be more impactful.

### Applying Emergence to Carcinogenesis

The emergence framework has been established through the creation of 12 principles. Each principle is presented in turn, followed by supporting evidence. Principles 1–10 encapsulate the key concepts upon which the framework is built and their application to carcinogenesis. Principle 11 relates the framework to cancer progression. The 12th principle relates to the application of the framework to translation research.

#### Principle 1

Cancer is a dynamic complex system emerging at the level of the ‘functional tissue unit’.

The first principle in creating an emergence framework of carcinogenesis is defining the ‘level’ at which ‘cancer’ as an emergent system arises. Cancer is characterized by alterations in cell and tissue structure, namely excessive accumulation of cells and disruption of normal tissue architecture (36, 54, 55). For some cancers, cell-level criteria are also of value, such as diagnostic nuclear envelope irregularity and chromatin clearing in early-stage thyroid papillary carcinoma (54). Hematological malignancies are diagnosed by assessing the proportion of immature precursor cells in bone marrow. Diagnosis is made using light microscopy and molecular analyses.

Analysis of genes alone does not make the diagnosis of cancer (25, 34). The presence or absence of genetic abnormality does not determine the presence of cancer: a ‘cancer’ gene may be present but not be expressed; not all malignancies have identifiable mutations; and the number of mutations is highly variable even within the same cancer type. Many normal cells can contain aberrations associated with cancer cells (1, 4, 36, 54).

**Figure 3:**
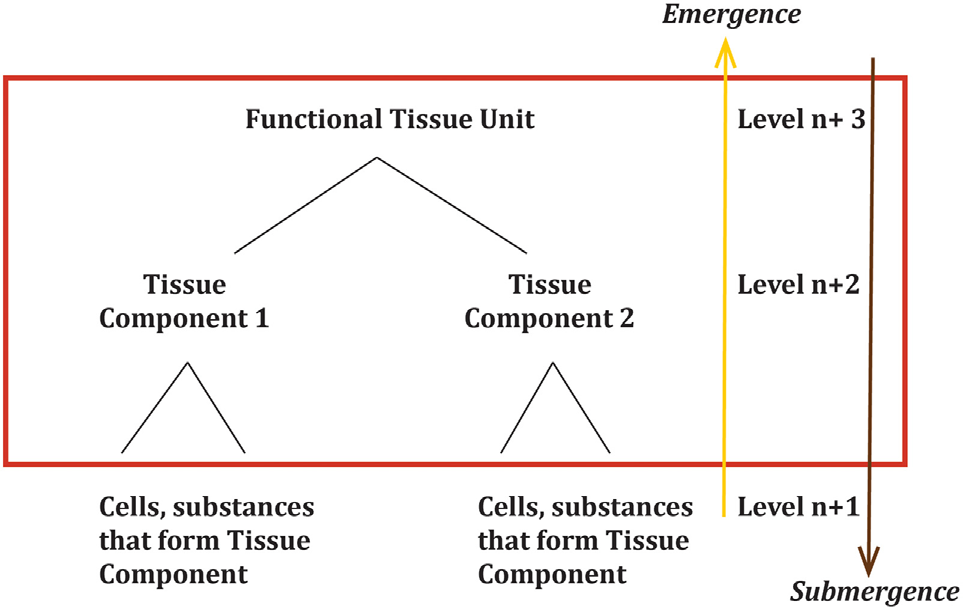
Cancer emerges as a dynamic system at the level of the ‘functional tissue unit’. The red box area highlights the levels of importance in an emergence framework. μ(s) or the ‘system’ is the ‘functional tissue unit’. The logical components C_a_(s) of the ‘functional tissue unit’ are the tissue components at the lower level.

Tissue-level patterns are diagnostic and tissue-level patterns may indicate mechanism (54, 55). It is this level at which cancer is diagnosed and it follows that this is the level that cancer emerges as a ‘system’. This level is not a single tissue type but a combination of tissues in a specific arrangement that have developed to form a system with specific properties and functions. These tissue level systems can be considered ‘functional tissue units’. Cancer, therefore, emerges at the level of ‘functional tissue units’ (56) (Figure 3) and can be defined as ‘disordered growth occurring at the level of functional tissue units causing changes of both morphology and physiology resulting in loss of normal function’.

This concept is supported by the work undertaken by Bissell (57, 58). Bissell and collaborators, based on experimental results of modeling both normal development of mammary glands and breast tumor formation, determined that the functional unit of the mammary gland is the mammary acinus, not simply the mammary epithelial cell and its extracellular matrix. Using a three-dimensional (3-D) culture system, they demonstrated the importance of the stroma in developing mammary acini and in augmenting function. This did not occur in 2-D models or cell lines. In contrast to normal breast cells, malignant breast cells did not form acini, but formed cell aggregates with large diameters and large number of cells, and did not produce casein (59).

Identifying the level at which cancer is diagnosed allows the construct of a normal ‘functional tissue unit’ system using a systems formula. The components of the system, C_a_(s), are the lower level ‘tissue components’ that combine in a specific self-organized structure, S_c_(s), to form the ‘functional tissue unit’.

**Figure 4:**
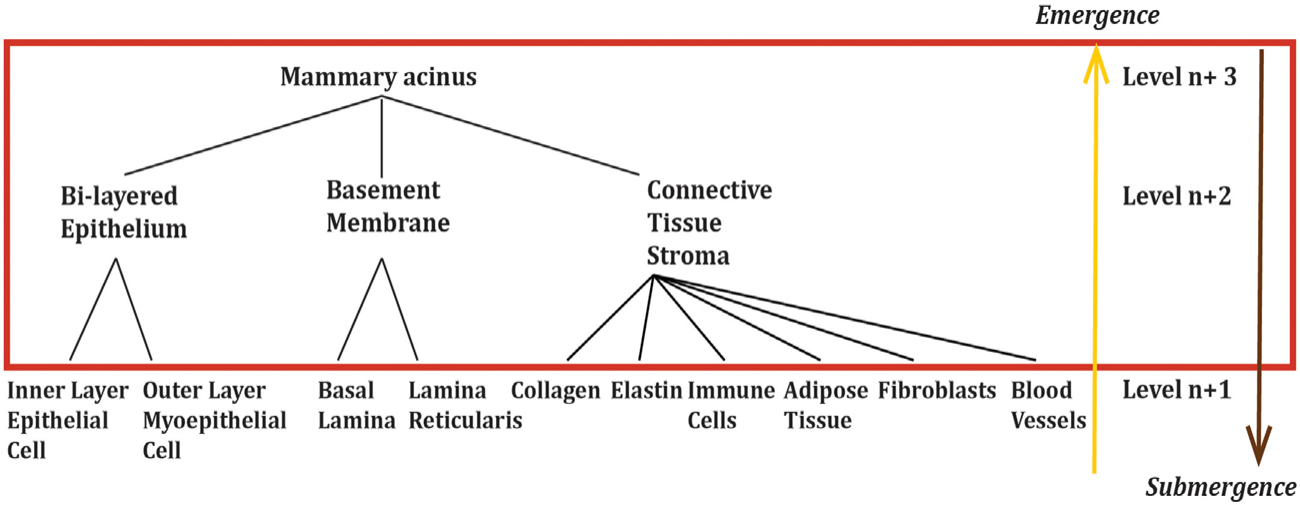
Mammary acinus as an example of a ‘functional tissue unit’, μ(s), the level at which ductal carcinoma as a system emerges. Again, the red box area highlights the levels of importance in an emergence framework. The logical components, C_a_(s), of the ‘functional tissue unit’ are the tissue components at the level below, Level *n* + 2. Components at lower levels are important only through the impact they have on the components, C_a_(s), by changing the components themselves and/or changing the structure, S_c_(s). Physiological changes alter the environment, E_b_(s), or mechanism, M_d_(s).

Placing ‘mammary acinus’ into an emergence model framework (Figure 4):

$$\mu_{\text{abcd}}(\text{s})=\{{\text{C}}_{\text{a}}(\text{s}),{\text{ E}}_{\text{b}}(\text{s}),{\text{ S}}_{\text{c}}(\text{s}),{\text{ M}}_{\text{d}}(\text{s})\}$$

then μ_abcd_(s) = functional tissue unit (mammary acinus) with the unique emergent functional property (to form glands capable of producing and releasing milk); C_a_(s) = the parts of the lower hierarchical level of tissue that combine to form the functional tissue unit (bi-layered epithelium, basement membrane, connective tissue stroma); E_b_(s) = the environment that interacts with the functional tissue unit or components of the functional unit (systemic hormones and factors in blood, external environmental factors *via* ducts); S_c_(s) = the specific arrangement and self-organization of the components of the functional tissue unit to form the functional tissue unit (bi-layered epithelium, basement membrane, connective stroma self-organize into a mammary acinus), the structure that enables interaction with the environment (blood vessels, epithelium); M_d_(s) = morphogenesis [collection of processes that enable formation of the functional tissue unit, including dynamic reciprocity (58)] and physiology (collection of processes that enable the functional tissue unit to perform its biological functions).

This principle enables research from both laboratory and clinical studies to be brought to a unified point through understanding the impact of findings on the various components of a normal ‘functional tissue unit’ that leads to its loss and replacement by an emergent cancer system.

#### Principle 2

Cancer is not a single disease entity, but an emergence phenomenon that can occur across numerous functional tissue units by multiple processes to generate a mechanism of carcinogenesis that is specific to that functional unit, and may be specific to an individual tumor; the common properties of cancer can be accomplished via different systems utilizing different mechanisms.

Different organs have different functional units and any one organ may have more than one functional unit. The emergence framework allows for each functional tissue unit to be considered as a unique ‘system’. The functional tissue unit of breast tissue is the mammary acinus. The pancreas has both an endocrine functional tissue unit and an exocrine tissue functional unit. The blood system has a functional tissue unit in the bone marrow, but the lymph glands could also be a functional tissue unit.

An example is ‘ductal breast cancer’. In an emergence framework, it would be considered to arise from the ‘components’ that form a ‘mammary acinus’. ‘Components’, ‘environment’, ‘structure’, and ‘mechanism’ enable the incorporation of the major theories of carcinogenesis into a unified concept. The “Hallmarks of Cancer”, as described by Hanahan and Weinberg (35, 60) are captured in ‘environment’ or ‘mechanism’. ‘Structure’ includes the key concept of TOFT as described by Sonnenschein and Soto (3, 13). Chronic inflammation (14) and evolutionary models are covered in ‘environment’ and ‘mechanism’, genetic modifications may alter ‘components’, ‘structure’, or be included in ‘mechanism’. The equation would be as follows.

μ_abcd_(s), the ‘mammary acinus’ system is replaced by μ_abcd_(sc), the cancer system emerging from the ‘mammary acinus’ system, ‘ductal carcinoma’

$$\mu_{\text{abcd}}(\text{sc})=\{{\text{C}}_{\text{a}}(\text{sc}),{\text{ E}}_{\text{b}}(\text{sc}),{\text{ S}}_{\text{c}}(\text{sc}),{\text{ M}}_{\text{d}}(\text{sc})\}$$

then μ_abcd_(sc) = ‘ductal carcinoma’ with loss of the property to form glands capable of producing and releasing milk and the new emergent properties of limitless replication, tissue invasion, and metastatic potential; C_a_(sc) = the parts of the lower hierarchical level of tissue that formerly combined to form the functional tissue unit (bi-layered epithelium, basement membrane, connective tissue stroma) in their altered form; E_b_(sc) = the environment that interacts with the ‘ductal carcinoma’ or components of the ‘ductal carcinoma’, which still include not only systemic hormones and factors in blood, external environmental factors *via* ducts, but also local tumor microenvironment and tumor-promoting inflammation; S_c_(sc) = the specific change in arrangement and self-organization of the components of the functional tissue unit to now form the ‘ductal carcinoma’, including altered polarity of epithelium, loss of normal tissue architecture, various stromal alterations, including angiogenesis, and loss of contact inhibition, breakdown of basement membrane, and invasion; M_d_(sc) = physiological processes that enable the formation of ‘ductal carcinoma’, including self-sufficiency in growth signals, insensitivity to anti-growth signals, evading apoptosis, sustained angiogenesis, deregulating cellular energetics, and avoiding immune destruction.

Comparing the systems equation for the relevant functional tissue unit to the systems equation of the cancer that has emerged from that specific functional tissue unit helps to identify the factors relevant for both the loss of the functional tissue unit system and emergence of the cancer system. For each functional tissue unit, the components, environment, structure, and mechanisms will vary. So too, will the specifics of the cancer that emerges from it.

This variation is seen at many levels, including genes. The functional tissue unit from which leukemia emerges is the bone marrow. In leukemia, *Notch1* is considered an oncogene. By contrast, the functional tissue unit from which oral squamous cell carcinoma emerges is the mucous membrane, and *Notch 1* is considered a tumor suppressor gene (61). Squamous cell carcinoma arising from skin and mucous membrane will have a similar phenotype but there can be a wide variety in mechanisms (62).

The overwhelming evidence supports that cancer is not just one aberrant cell or one disease, but many (1, 4). Identifying from which functional tissue unit a cancer has emerged is key.

#### Principle 3

Causation of cancer is a property of the system and is not contributable at any single hierarchical level: multiscale causality associates causation at different levels concomitantly.

As can be deduced from Figure 3, significant changes in even lower levels could impact the emergence of one or more of the tissue components, which would in turn impact the emergence of the ‘functional tissue unit’. This shows the path of upward causation. However, as the relationship between levels is not linear, a single change in a single cell is highly unlikely to generate a change at the level of the ‘functional tissue unit’. For a system property to emerge or submerge, a point of criticality must be reached. This is discussed further below.

As opposed to evolutionary models, an emergence framework is not required to start at the genetic level, nor does the process need to be gradual or stepwise. An emergence framework allows for discontinuity and major rapid transformation in state.

Changes in S_c_(s) that impact the exostructure or the way the system interacts with the environment, E_b_(s), can occur as a direct response to changes in E_b_(s), as a result of changes to C_a_(s) impacting S_c_(s), and/or the output of the system, μ_abcd_(s), directly causing changes in E_b_(s).

The output of the system, μ_abcd_(s), may also create feedback affecting lower levels. This is downward causation.

The mechanism, M_d_(s), or group of processes by which the ‘functional tissue unit’ achieves its emergent property or function, can also be impacted by upward causes, downward causes, and environmental causes. Physiological changes are as important as morphological ones.

Causation can flow in many directions simultaneously, there is no single privileged level of causation, and causation is not necessarily linear (15, 17, 22, 37, 38, 63).

#### Principle 4

The ‘state’ of a cell is determined by its position within the functional tissue unit and the state of the functional tissue unit as a system. All living systems metabolize and are therefore dynamic over time. Defining a ‘default’ state of a cell as quiescent or proliferative is not relevant in an emergence framework of carcinogenesis.

All living tissues metabolize and, therefore, create dynamic open systems experiencing various iterations of pendulum-like swings in their morphology and physiology, which are controlled by resetting mechanisms. These are the oscillations or ‘clocks’, and molecular motors referred to in the preamble (29, 50–53). For each cycle, there is a defined beginning and an end. Cycles express relatedness, a key characteristic of systems, implying the interconnectedness and dependability of all components (18, 39).

The ‘state’ of cells (quiescent or proliferative) is largely shaped by their roles and positions within their community (7). Mapped in a state-space over time, changes would be seen in the variables that compose the system in response to inputs from its environment and the outputs it puts back and other internal changes. Fluctuations occur in order to maintain the function of the ‘functional tissue unit’ in accordance with the natural law of that state. Therefore, there can be no ‘default state’ of a cell in a system, as it is never free of the system it is part of (7). If a cell became a closed system, that is it has ceased to communicate with its environment, it would move into a state of entropy and cease to exist (18).

Evidence that cells *in vivo* function as a community can be found in the science laboratory. Placing cells in culture disrupts tissue architecture and cell–cell relationships, allowing the emergence of cells with malignant potential, that is, those that can grow and replicate autonomously (55). Conversely, growing cells in Matrigel, the extracellular matrix produced by Engelbreth–Holm–Swarm sarcoma cells allows the growth of 3-D structures that resemble normal tissue, and supports, for example, the branching, morphogenesis in mammary tissue that produces duct-like structures and responds to lactogenic hormones (55, 57, 59).

Investigation into morphogenesis, morphogens, and morphostats provides additional evidence for the position within functional tissue units determining the ‘state’ of any given cell (55, 56). Morphogens are fundamental organizers of tissue morphology and play a critical role in embryogenesis. Morphastats are believed to have a central role in maintaining normal cellular behavior and microarchitecture in adult tissues. Both are substances to which cells respond directly, but there are two or more qualitatively different responses depending on the concentration and, therefore, the distance from, and by extrapolation, the relative position to the source (55).

An example is the regular controlled migration of basal layer to surface layer in adult epithelial tissues. Each epithelial cell passes through several stages: a reproductive transit cell located basally; an intermittent cell with functional capacity located centrally; and a quiescent/senescent cell or cell remnant located at the surface (55). Position determines the state of the cell.

#### Principle 5

A healthy functional tissue unit is a metastable system oscillating between maintaining optimum function, maximal adaptability in response to inputs and outputs with its environment and self-maintenance through repair, differentiation and apoptosis in accordance with the natural law of a functional tissue unit as defined by

$$F=\frac{k}{M}$$

where *F* = functional status of the functional tissue unit; *M* = repair/growth rate; *k* = non-zero constant.

**Figure 5:**
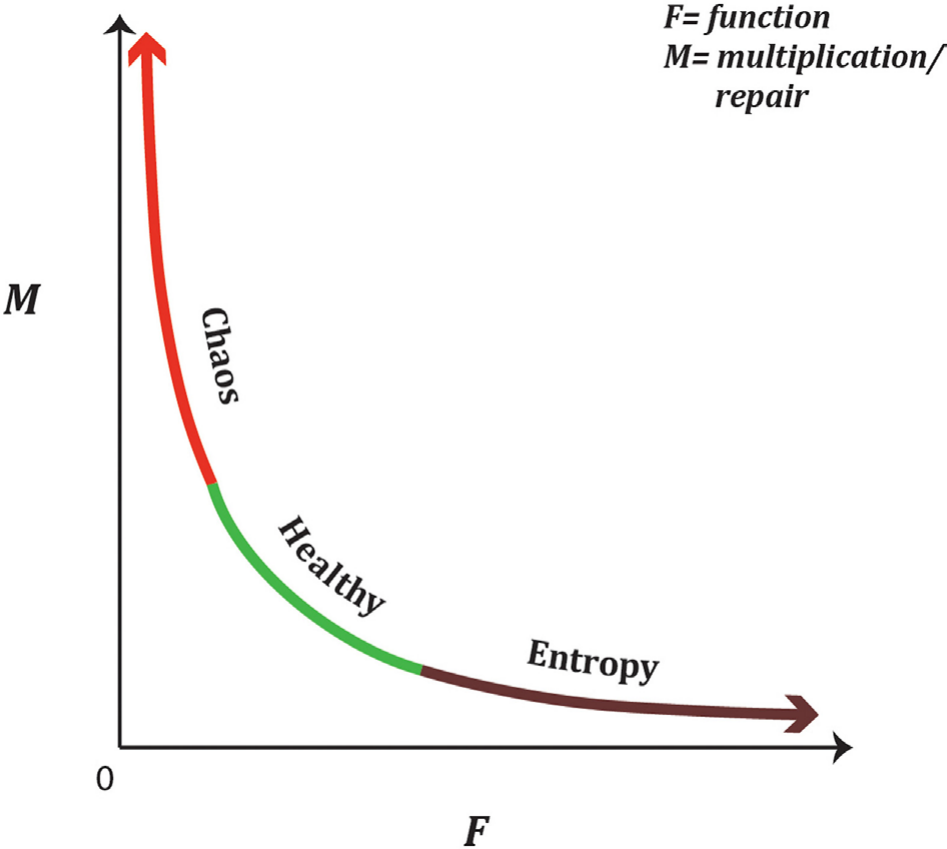
A functional tissue unit is a metastable dynamic system oscillating between maintaining optimum function, maximal adaptability in response to inputs and outputs with its environment, and self-maintenance through repair, differentiation, and apoptosis. The natural law of a functional tissue unit is an inverse non-linear relationship between function, ‘F’, and growth/repair, ‘M’. This is represented graphically, by the equation $F=\frac{\text{k}}{M}$ with ‘k’ being a non-zero constant. If the functional tissue unit swings too far toward optimal function, it reduces its ability to adapt to environmental changes and will ultimately become a closed system with inability to gain energy from its surrounds and in accordance with the second law of thermodynamics will move toward entropy (aging and death). A functional tissue unit that undergoes excessive growth or repair is redirecting energy away from normal function and increases the need to generate additional usable energy from its environment, pushing it into the hyper-energized state.

A functional tissue unit is a dynamic system that is constantly re-setting mechanisms to allow for complex adaptability in balancing inputs and outputs in response to internal and environmental changes, to maintain stable boundaries, and to maintain its function. A healthy functional tissue unit sits in a metastable state oscillating between order and the inner edge of chaos, undergoing cellular renewals, cellular differentiation, and cellular apoptosis as required by the functional tissue unit, maintaining immunosurveillance and controlling angiogenesis (18, 29, 46). The natural law of a functional tissue unit is a reciprocal relationship between functionality and repair/multiplication and can be represented graphically as in Figure 5.

Various stimuli can alter the dynamic state of a functional tissue unit moving it toward a higher rate of growth and/or repair, such as in wound healing, with changes in motility, polarization, matrix formation, and expression of various proteins and cytokines (64–67) shifting the oscillation of the functional tissue unit more toward chaos. Alternatively, a shift toward increased function results in a functional tissue unit losing its complexity and ability for renewal (41, 68). This may manifest in the functional tissue unit becoming non-responsive to its environment and functioning autonomously, such as parathyroid glands in primary parathyroidism (69) or involution due to entropy such as in Type 2 diabetes (41, 68).

In carcinogenesis, precancerous states represent a shift of the oscillating metastable state of the functional tissue unit toward one of these two extremes.

#### Principle 6

A functional tissue unit will have points of self-organized criticality in both the directions of entropy and chaos, beyond which a critical collapse occurs, resulting in loss of the morphological and physiological properties of the functional tissue unit setting the initial state from which cancer can emerge.

In line with Principle 5, if a functional tissue unit becomes too stable, adaptability and, therefore, resilience to environmental changes is lost, leading to entropy, or degeneration (18, 70). A system that becomes too chaotic will lose its structure and function and be pushed toward the development of a new state. At each extreme of the ‘healthy’ metastable state, there is a point of self-organizational criticality that when surpassed results in the collapse of the next hierarchical level. At or near this point, a change may occur that in itself is insignificant, but in the system can lead to a massive, rapid, and seemingly disrupted transformational occurrence (7, 18).

Therefore, in this framework, a change or combination of changes in C_a_(s), E_b_(s), S_c_(s), or M_d_(s), beyond a point of self-organized criticality in either direction, alters the system causing disruption of the bonding structure that enables self-organization of the collection of relations among the biological tissue components, and/or their environment and/or the group of processes producing the mechanism of morphogenesis. This results in loss of the morphological and physiological properties of the functional tissue unit. Once this occurs, there is an opportunity for a new system, namely ‘cancer’ to arise.

The work of Nordemar et al. (71) in which they assessed progression of cancer *in situ* (CIS) in the larynx to invasive carcinoma can be used as an example of how this principle may be applied. While not all CIS lesions staining positive for the laminin γ2 chain of laminin 332 (formally laminin 5) progressed to invasive cancer, *only* lesions expressing laminin 332 progressed. In this framework, these results could be interpreted that the expression of laminin 332 was an indicator of the point of self-organized criticality.

#### Principle 7

Reduced redundancy of healthy functional tissue units through either entropy (degeneration) or excessive repair in response to tissue trauma is associated with an increased risk of cancer.

‘Redundancy’ is another key feature of system resilience or robustness (18, 25). Redundancy of functional tissue units and redundancy within the tissue components of the functional tissue units are important considerations in an emergence framework of carcinogenesis.

If the metastable state passes too far toward entropy, there is an increasing inability to adapt to the environment, and the functional tissue unit moves from being an open system toward an increasingly closed one. Increased entropy results in inadequate useable energy within the functional tissue unit to maintain function and structure (18, 70, 72, 73). At a higher hierarchical level, the organ may shut down and rid itself of an entropic functional tissue unit that has passed the point of self-organized criticality toward entropy, in the same manner that a functional tissue unit may trigger apoptosis of a cell to maintain the health of the system (18, 63, 72–74).

Aging has been shown to be associated with breakdown of non-linear dynamics and fractal patterns with subsequent loss of complexity in numerous areas, including liver metabolism, cardiac physiology, and gait (75–78). Degeneration that occurs with aging leads to loss of redundancy through reduction of healthy functional tissue units available to maintain the overall function of the organ (63, 70, 74, 79). This has a number of important consequences. Numerous functional tissue units having such reduced interaction with their environment may become perceived as ‘foreign tissue’, triggering an immune response, manifesting in an autoimmune disease (18). There are a lower number of healthy functional tissue units to respond to requirements of repair, and less capacity to remove functional tissue units that have swung too far in the other direction into the chaos zone. Finally, the entropic functional tissue units may not be able to respond to the requirements of repair in a healthy manner due to decreased interaction from the environment, and die or attempt to undergo repair and growth that is disordered. Resilience protecting against the emergence of cancer is, therefore, increasingly lost with age (70, 72–74). The same age incidence curves used to propose and support SMT are consistent with an emergence framework of carcinogenesis.

Moving to the other extreme, if a functional tissue unit is required to undergo excessive repair or growth, this could push it past the self-criticality point into chaos. ‘Trauma’ in all its manifestations, including inflammation, mechanical trauma, carcinogens, and infective causes, would push a functional tissue unit toward repair. As the natural law of the functional tissue unit is that function is inversely proportional to repair/multiplication, this will be the point where the law can no longer be maintained and the functional tissue unit as a system collapses. Loss of tissue organization architecture would also result. An emergence framework is, therefore, supported by the same evidence that supports TOFT as proposed by Sonnenschein and Soto (12), and the evidence that supports the inflammatory paradigm proposed by Brücher and Jamall (14).

#### Principle 8

Risk factors for cancer act to reduce redundancy of functional tissue units via a number of mechanisms at a faster rate than in the natural aging process, generating an increased risk for cancer.

Individuals who are exposed to various risk factors such as smoking, excessive alcohol intake, stress, exposure to radiation, and other life style insults, prematurely reduce the redundancy of the functional tissue units of various organs, reducing resilience to aging processes (73). Consequently, they have higher risk of various diseases as they become older, including cancer. Environmental insults that produce repeated or persistent tissue ‘trauma’ reduce healthy functional tissue units by pushing the system into a state of repair, and beyond, also impact on functional tissue unit redundancy. Hereditary factors or genetic profiles associated with increased cancer rates alter baseline redundancy through increased entropy or excessive repair. Precise mechanisms are wide and varied (72, 73, 80–82).

An emergence framework of carcinogenesis provides a common system end-point at the level of the functional tissue unit, reduced redundancy, and hence resilience and is consistent with the multivariate findings for risk factors linked to the majority of cancers.

#### Principle 9

Loss of the properties that make a functional tissue unit a ‘system’ is a prerequisite to create the ‘initial state’ for the emergence of cancer.

Prior to a new system emerging, a pre-system or ‘initial state’ exists. This is a point where potential components become randomly associated rather than being combined in a self-organized structure. Potential exists for the emergence of new systems (18). In carcinogenesis, this is the point where functional tissue units move so far along entropy or chaos vectors that there is a breakdown in structure and loss of function of functional tissue units, leaving only an association of tissue components with the potential to combine into a new form. At the chaos extreme, it is the excessive energy being acquired to drive repair or growth that tips the scale. At the entropy extreme, there is insufficient available energy to maintain structure and function, which causes the system to break down, releasing its tissue components to the state of chaos of the lower level. It is from these points that cancer has the opportunity to arise as an emergence phenomenon. Cancer cannot arise from healthy functional tissue units.

#### Principle 10

‘Cancer’ is the emergence of a new ‘system’ arising from the tissue components of a functional tissue unit that has lost its normal self-organization arrangement and function, identifiable by changes in morphology and physiology.

The events that occur in this ‘initial state’ are critical in determining the potential outcome, including apoptosis or involution, clearing by the immune system, formation of scar or fibrosis, stabilization and reformation of functional tissue units, or the formation of cancer.

Cancer has commonly been described as a state of disorder and ‘chaos’. However, using the definition of ‘chaos’ as previously described, and the exquisite sensitivity to ‘initial condition’, this would imply that the likelihood of ever seeing two cancers at the same time would be extremely small (18, 40, 46). In reality, this is not the case. Over time, a specific type of cancer exhibits repetitive and largely predictable patterns in numerous patients. For example, breast cancer will most commonly spread to the axillary nodes, before spreading to other tissues, including bone and liver. Head and neck squamous cell cancer will spread to regional nodes before metastasizing to lungs. Papillary thyroid cancer spreads to the local neck nodes first, whereas follicular thyroid cancer may spread elsewhere in the body. This supports that cancer is not simply random disorganized cell multiplication, but a complex, dynamic, and evolving system. Further evidence to support this theory is the identification of fractal patterns appearing in both cancer cells and tumors (42, 83–86).

The importance of considering cancer as a system is that the components will include *all* the components that previously formed a healthy functional tissue unit. This means, for example, in solid epithelial tumors, such as head and neck squamous cell carcinomas, the ‘system’ includes not only the abnormal epithelial cells but the stroma as well. The importance of stroma and microenvironment is well supported by the work of many researchers (57, 59, 87–93). An emergence framework of carcinogenesis incorporates these findings.

#### Principle 11

Cancer progression is the deterministic development of a sequence of rapidly adapting emergent systems, each with identifiable patterns of morphology, physiology and behavior, the dynamics of which can be studied via a state-space approach.

Defining cancer as a system in an emergence framework also enables cancer progression to be seen as a sequence of rapidly adapting emergent systems, each of which will have identifiable patterns of morphology, physiology, and behavior. Despite the likely multiple mechanisms that initiate cancer, there is a surprisingly stereotypical and deterministic pattern of cancer. Epithelial cancers follow a general pattern of growth, dysplasia, invasion, local metastases, distant metastases, and ultimately destruction of the host. Patterns of progression are seen too in sarcomas and hemopoetic cancers (29, 44, 48, 86).

The primary driver of functional tissue units toward chaos is the need for growth and repair, either in an attempt to self-salvage from entropy or in response to environmental factors. These processes require an increase in energy. This energy needs to be brought into the system from the environment (29). A persistent requirement to repair or grow will result in the need for an ongoing increased level of energy within the system. In line with Principle 6, there is a point of self-organizing criticality where the attempt of the functional tissue unit to persistently maintain higher energy levels will push it into chaos and enable the emergence of a new system with new complexity.

Evidence for this is multifaceted. In cancer cells, there is an increase in glycolysis (the Warburg effect) (29, 94–96). This phenomenon has been utilized clinically with the development of PET scans that use radiolabeled glucose analog 18-fluorodeoxyglucose to detect radiologically the higher rate of glucose metabolism found in malignancy (94–96). Originally proposed to be due to mitochondrial respiration defects, this has now been linked to oncogenic driver mutations, such as activation of K-rags, c-Myc, and phosphatidylinositol-3 kinase, or loss of phosphatase and tensin homolog (Pten) and p53, further increasing energy production through glycolysis in addition to, rather than instead of, mitochondrial respiration (95).

Mitochondria are responsible for about 80% of normal cellular energy (29). In cancer cells, mutations have been noted in mitochondrial DNA; however, importantly, there is a lack of accumulation of mitochondrial genome errors. The errors noted do not inactivate energy metabolism, but appear to alter the bioenergetic and biosynthetic state of the cell (95, 96). Moreover, there is a strong selective pressure to retain and accumulate respiration functional mitochondria in malignant tumors, with active quality control of mitochondria through mitophagy, preventing accumulation of defective mitochondria and release of the substrates for reuse.

1–5% of oxygen consumed will produce reactive oxygen species (ROS), or free radicals (29). In cancer cells, increased demand on mitochondria to produce energy is reflected in increased ROS (29, 95, 96). High ROS production can be toxic to a cell (94–96). Increased ROS production when apoptosis is inhibited, however, changes the cell’s redox status, altering activities of transcription factors, such as hypoxia-induced factor (HIF-1α) and FOS–JUN heterodimer, which constitute an AP–1 transcription factor, and lead to histone and DNA methylation, stimulating cancer proliferation (95, 96). Cancer cell ROS production inactivates caveolin 1 in adjacent stromal fibroblasts. This increases mitophagy, reduces mitochondrial function and, increases lactate production in these fibroblasts. Secreted stromal cell lactate then fuels cancer cell oxidative metabolism, which drives tumor growth and proliferation (96).

Angiogenesis is an identified hallmark of cancer. Recent data indicates that angiogenesis contributes to both the microscopic premalignant phase and ongoing phases of neoplastic progression (35). During tumor progression, an ‘angiogenic switch’ is almost always activated and remains on, causing normally quiescent vasculature to continually sprout new vessels, increasing nutrition and oxygen supply to tumors, and fueling energy needs that help sustain expanding neoplastic growths (35, 97). HIF-1α is a key regulator of hypoxia-induced angiogenesis in tumors (97).

Cachexia, which is characterized by weight loss and inflammation, is present in 40–80% of cancer patients, depending on tumor type, and is associated with metabolic changes involving carbohydrate, lipid, and nitrogen metabolism. These changes are linked with the need to maintain glycemia and to sustain tumor growth (98).

The observation of fractals emerging at various stages of tumor progression is also supportive of viewing cancer progression as a sequence of emergent systems. As described in the preliminary description of chaos, fractals occur in space-chaos and their appearance is reflective of deterministic development with emergence of a new system with new complexity (42, 45, 47, 85, 86).

Fractal patterns have been observed in the neo-angiogenesis patterns associated with tumors (43, 45). Cancer-specific fractal geometry has been found at the tissue level when analyzing tumor perimeters (48, 84). Recently, fractal patterns were observed with atomic force microscopy to appear on the cell surface as premalignant cells transformed into malignant cells in cervical epithelial cells (47).

Applying the systems formula, an emergence framework enables each ‘stage’ in the progress of cancer (invasion, local metastases, distant metastases, and destruction of the host) to be considered as a new emergent property. Using the state-space methodology, (*F*(t) = {X(t), Y(t)}) the dynamics of each stage can be studied using data and information relevant to that stage and to the level at which investigation is being focused. Patterns can be observed, investigated, and ultimately provide guidance in management decisions.

#### Principle 12

An emergence framework of carcinogenesis provides a common united framework for facilitating and integrating cancer research across all areas of basic, clinical and translational research by directing focus to a common level at which the diagnosis of cancer is made, the functional tissue unit.

An emergence framework of carcinogenesis provides a framework for translational research that is consistent and workable with the definitions of the European Society of Translational Research, the National Institutes of Health (USA), Australia’s National Health and Medical Research Council, and the UK’s Medical Research Council. It enables the starting point to be anywhere in the system as it directs all starting points to a common level, that of the functional tissue unit. The interplay of laboratory observations, clinical outcomes, genetic studies, community patterns, or therapeutic trials can be assessed within this framework by considering how they impact on the systems equation of the functional tissue unit and/or the emergent cancer.

Having a common framework provides a consistent format for investigating, allows for cross-referencing, enables integration, and facilitates the use of biosystematics and computational modeling.

## Conclusion

Living systems are dynamic, non-linear, and complex. Increasingly, biology is recognized as its own discrete discipline that cannot be adequately explained by reducing living processes to their physicochemical properties.

Viewing cancer as a complex emergent system creates a new perspective that moves away from the gene-centric reductionist approach that has been the predominant theory of carcinogenesis for more than half a century. Through the integration of concepts from systems biology, physics, chemistry, and various theories of carcinogenesis, a new practical emergence framework of carcinogenesis has been proposed, based on 12 key principles. These are summarized in Table 3.

**Table 3:** An emergence framework of carcinogenesis: the 12 Principles.

Principle 1	Cancer is a dynamic complex system emerging at the level of the ‘functional tissue unit’

Principle 2	Cancer is not a single disease entity, but an emergence phenomenon that can occur across numerous functional tissue units by multiple processes to generate a mechanism of carcinogenesis that is specific to that functional unit and may be specific to an individual tumor; the common properties of cancer can be accomplished *via* different systems utilizing different mechanisms

Principle 3	Causation of cancer is a property of the system and is not contributable at any single hierarchical level: multiscale causality associates causation at different levels concomitantly

Principle 4	The ‘state’ of a cell is determined by its position within the functional tissue unit and the state of the functional tissue unit as a system. All living systems metabolize and are, therefore, dynamic over time. Defining a ‘default’ state of a cell as quiescent or proliferative is not relevant in an emergence framework of carcinogenesis

Principle 5	A healthy functional tissue unit is a metastable system oscillating between maintaining optimum function, maximal adaptability in response to inputs and outputs with its environment, and self-maintenance through repair, differentiation and apoptosis in accordance with the natural law of a functional tissue unit as defined by: $F=\frac{k}{M}$ where *F* = functional status of the functional tissue unit, *M* = repair/growth rate, *k* = non-zero constant

Principle 6	A functional tissue unit will have points of self-organized criticality in both the directions of entropy and chaos, beyond which a critical collapse occurs, resulting in loss of the morphological and physiological properties of the functional tissue unit setting the initial state from which cancer can emerge

Principle 7	Reduced redundancy of healthy functional tissue units through either entropy (degeneration) or excessive repair in response to tissue trauma is associated with an increased risk of cancer

Principle 8	Risk factors for cancer act to reduce redundancy of functional tissue units *via* a number of mechanisms at a faster rate than in the natural aging process, generating an increased risk for cancer

Principle 9	Loss of the properties that make a functional tissue unit a ‘system’ is a prerequisite to create the ‘initial state’ for the emergence of cancer

Principle 10	‘Cancer’ is the emergence of a new ‘system’ arising from the tissue components of a functional tissue unit that has lost its normal self-organization arrangement and function identifiable by changes in morphology and physiology

Principle 11	Cancer progression is the deterministic development of a sequence of rapidly adapting emergent systems, each with identifiable patterns of morphology, physiology and behavior, the dynamics of which can be studied *via* a state-space approach

Principle 12	An emergence framework of carcinogenesis provides a common united framework for facilitating and integrating cancer research across all areas of basic, clinical, and translational research by directing focus on a common level at which the diagnosis of cancer is made, the functional tissue unit

The motivation in developing an emergence framework of carcinogenesis was to create a common, comprehensive, and integrative framework for researchers, clinicians, philosophers, thought leaders, institutions, and funding bodies involved in researching and treating cancer. The authors have aimed to collate and synthesize current concepts and evidence around carcinogenesis into a single framework that incorporates previously incompatible viewpoints and ideas.

It would not have been possible to construct this framework without the work of the significant body of researchers, scientists, clinicians, and philosophers who have already contributed to this field. Accordingly, the reference list is limited to key publications.

Max Planck, a German Physicist known as the founder of Quantum Physics and recipient of the Nobel Prize for Physics in 1918, is quoted as saying:

“When you change the way you look at things, the things you look at change”.

We hope that this ‘emergence framework of carcinogenesis’ challenges the reader to look at cancer and carcinogenesis differently.

## Author Contributions

ES, the primary author, was responsible for the concept, development of the framework, drafting, revising, and finalizing of the article. BW contributed to the development of the concept, provided critical feedback with suggested revisions, and was involved in finalizing the article. Both ES and BW agreed to be accountable for all aspects of the work in ensuring that questions related to the accuracy or integrity of any part of the work are appropriately investigated and resolved.

## Conflict of Interest Statement

The authors declare that the research was conducted in the absence of any commercial or financial relationships that could be construed as a potential conflict of interest.
